# Effects of Resveratrol on *FOXO1* and *FOXO3*a Genes Expression in Adipose Tissue, Serum Insulin, Insulin Resistance and Serum SOD Activity in Type 2 Diabetic Rats

**DOI:** 10.22088/IJMCM.BUMS.7.3.176

**Published:** 2018-12-31

**Authors:** Soheila Asadi, Zohreh Rahimi, Massoud Saidijam, Nooshin Shabab, Mohammad Taghi Goodarzi

**Affiliations:** 1 *Department of Clinical Biochemistry, Faculty of Medicine, Kermanshah University of Medical Sciences, Kermanshah-Iran.*; 2 *Research Center for Molecular Medicine, Hamadan University of Medical Sciences, Hamadan, Iran.*

**Keywords:** Oxidative stress, diabetes mellitus, FOXO, insulin resistance, resveratrol, superoxide dismutase

## Abstract

Induced oxidative stress in diabetes mellitus (DM) plays a critical role in insulin resistance. Fork head-related transcription factor (FOXO) proteins are important transcriptional factors involved in oxidative stress and insulin resistance. Resveratrol (RSV) is a polyphenol with hypoglycemic and antioxidant properties. The aims of the present study were to examine the effects of RSV on *FOXO *gene expression, serum superoxide dismutase (SOD) activity, insulin level, and insulin resistance in type 2 diabetic (T2DM) rats. Thirty male Wistar rats were used in this study. DM was induced in rats (n=24) using streptozotocin (STZ) and nicotinamide; then, they were divided into 4 groups of 6 rats each. Six untreated normal rats were used as normal control group; diabetic rats in groups 2 to 5 were treated with 0, 1, 5 and 10 mg /kg body weight of RSV, respectively for 30 days. At the end of the experimental period, the rats were sacriﬁced, their sera were separated, and adipose tissues were obtained and stored at −80 °C. Serum glucose and SOD activity levels were determined biochemically, and serum insulin level was determined by ELISA method. Gere expression in *FOXO1* and *FOXO3a* in adipose tissue was evaluated using real‐time PCR. Results indicated that RSV significantly reduced blood glucose level, increased insulin level and improved insulin sensitivity. RSV resulted in an increased serum SOD activity and caused decreased *FOXO1* and *FOXO3a* expression in adipose tissue of rats with T2DM. Therefore, by attenuation of *FOXO* expression in adipose tissue of T2DM rats, RSV showed a hypoglycemic potential and antioxidant properties, and consequently ameliorated insulin resistance.

Diabetes mellitus (DM) is one of the most important metabolic disorders with impaired glucose, fat and protein metabolism, which contribute to a high morbidity and mortality worldwide (1). DM results in hyperglycemia due to defect in insulin secretion and/or insulin resistance (2). Hyperglycemia induces reactive oxygen species (ROS) production, leading to oxidative stress that plays a critical role in DM- associated complications such as nephropathy, retinopathy and cardiovascular disease (1, 3, 4). Oxidative stress may lead to insulin resistance through several mechanisms. One of these mechanisms is the activation of forkhead box-related (FOXO) transcription factors (5). The FOXO family includes four transcription factors (FOXO1, FOXO3a, FOXO4 and FOXO6) that control the expression of genes involved in DNA repair, apoptosis, metabolism, oxidative stress, insulin resistance and longevity (6, 7). The FOXO proteins play an important role in the metabolism, and are expressed in all tissues especially in adipose tissue, heart, brain, liver and skeletal muscle (5). These proteins play a significant role in insulin resistance and oxidative stress and their activity is regulated by silencing information regulators (sirtuins, SIRTs) (5). Deacetylation of this protein by SIRTs results in phosphorylation and inactivation of this transcription factor by SIRTs (8, 9). Therefore, by affecting SIRTs (inactivation) DM results in acetylation and activation of FOXO and subsequent hyperglycemia, oxidative stress and insulin resistance. Thus, the activators of SIRTs or inhibitors of FOXO may improve hyperglycemia and oxidative stress and consequently insulin resistance (5). Today, herbal medicine has a particular role in disease treatment. Polyphenolic compounds such as resveratrol (RSV) has a beneficial effects on DM. RSV (3, 5, 4’-trihydroxystilbene) is a polyphenol found in *Polygonum cuspidatum*, red grapes, red wine and berries (10). Many studies indicated that RSV has a hypoglycemic, anti-inflammatory and antioxidant effect (11, 12). It has been established that RSV is a potent activator of SIRT1 and the activation of SIRT1 attenuates hyperglycemia (13). Additionally, due to the effect of RSV on SIRT1 activity, it can be concluded that RSV might have a beneficial effect on *FOXO1*, and *FOXO3a* expression in adipose tissue, and might consequently improve the oxidative stress and insulin resistance. The aim of the present study was to examine the effect of RSV on blood glucose level, *FOXO1* and *FOXO3a* expression in adipose tissue, insulin level, insulin resistance and serum superoxide dismutase (SOD) activity in rats with T2DM.

## Materials and methods

**Animals and study design**

Thirty male Wistar rats (6-8 weeks old, weighing 150-200 g) were used in the present study. Rats were purchased from Razi Institute, Iran, and maintained in the central animal house, Hamadan University of Medical Sciences (Hamadan, Iran). The rats were housed in standard plastic cages (4 rats per cage) under a standard condition (12 h light and dark cycle, temperature 22±2 ˚C) and free access to water and standard chow diet. All experiments were conducted in accordance with the National Institutes of Health guidelines on animal care (14). The Research Committee of Hamadan University of Medical Sciences (Hamadan, Iran) approved the research procedures. The study was carried out according to “Guide for the care and use of laboratory animals”, and the Ethics Committee of Hamadan University of Medical Sciences approved the experimental protocols. The rats were divided into five groups based on simple randomization method (15). The RSV was given orally (gavage, suspension) to the experimental groups at three different doses i.e. 1, 5 and 10 mg/ kg of body weight/ day (mg/kg.bw/day). Five groups (six rats in each) were designed as follows: a normal control group (healthy control); a diabetic control group (untreated diabetic); and three diabetic + 1, 5, and 10 mg/kg.bw/day RSV, respectively groups.

The glucose level was measured using a glucometer on day zero (D0) before induction of T2DM. For induction of T2DM in diabetic rats, the overnight fasted rats were injected intraperitoneally (IP) 60 mg/kg.bw streptozotocin (STZ, Sigma, in 0.1 M sodium citrate pH 4.5) followed by 120 mg/kg.bw nicotinamide (NA, Sigma) after 15 min (16). The normal control group received the same volumes of 0.1 M sodium citrate buffer as carrier. To confirm the T2DM, 72 h after induction of diabetes, glucose level was assessed using a glucometer (Accuchek, Roche, Germany). The rats with blood glucose level higher than 150 mg/dl were considered as having T2DM (17). Seven days after T2DM induction, diabetic case groups, received 1, 5, and 10 mg/kg.bw/day doses of RSV, respectively. RSV was suspended in deionized water and administered using gavage at 10 am each day for 30 days. At the end of treatment period, the rats were anesthetized using ketamine: xylazine (100 mg/kg.bw : 5–10 mg/kg.bw, IP) and sacrificed (18). Blood sample was collected by cardiac puncture; serum was separated and stored at -20 °C. Visceral adipose tissue was separated from each rat, cut into small pieces and was immediately frozen in liquid nitrogen and stored at -80 °C until analysis.

**Determination of biochemical parameters in serum**

The serum glucose level at day 37 was assessed by glucose oxidase method using a biochemical kit (Pars Azmun, Iran). Insulin was measured using rat insulin ELISA kit (Alpco, USA). Insulin resistance index (HOMA) was calculated by using “insulin (μU/ml) × glucose (mmol/l)/22.5” formula (19). The serum SOD activity was measured using a biochemical kit (Randox, England) and expressed as unit/ml.

**RNA extraction and RT-PCR**

The RNA extraction from adipose tissue was performed manually using TRIzol (Invitrogen, USA) according to manufacturer’s protocol. RevertAid first strand cDNA synthesis kit (Thermo scientific, USA) was used for cDNA synthesis using 1 µg of RNA. The SYBR Premix Ex Taq II (TaKaRa, Japan) was used to amplify the cDNA on a CFX96 Real-Time PCR detection system (BioRad, USA) and determine *FOXO1 *and *FOXO3a* mRNA expression levels. The primers were designed by AlleleID6 software (Premier Biosoft Corporation, USA), and their sequences are listed in Table 1. The relative copy number of each gene was determined and normalized to the amount of 18S RNA as housekeeping gene. Gene expression fold change was then calculated by 2^-ΔΔCT^ formula.

**Statistical analysis**

Statistical analysis was carried out using the Statistical Package for Social Sciences version 16 (SPSS Inc., Chicago-USA). The one-way ANOVA with post hoc Tukey test was used for comparison between groups. Data are presented as mean±SD and P values less than 0.05 (P<0.05) was defined as statistically significant. The correlation between variables (*FOXO3a*, *FOXO1*, SOD activity and HOMA) was tested using Pearson’s correlation coefficient and chi square test.

## Results

**Effect of RSV on blood gl** **u** **cose levels, insulin**

**levels, and insulin resistance**

As indicated in Table 2, there was no significant difference in blood glucose levels between different studied groups at D0 (before induction of T2DM). Nevertheless, data from D37 (after completion of the treatment period) showed higher blood glucose levels in diabetic control group and diabetic rats that were treated with different doses of RSV in comparison with normal control group. Furthermore, treatment with 5 and10 mg/kg.bw/day of RSV caused a significant reduction in blood glucose level in comparison with diabetic control group. Table 2 demonstrates that insulin level significantly decreased in diabetic control group and diabetic group treated with 1 mg/kg.bw/day of RSV. In addition, insulin resistance (HOMA) in untreated diabetic group and diabetic group treated with 1 mg/kg.bw/day of RSV significantly increased in comparison with normal group. However, treatment with 5 and 10mg/kg b.w/day of RSV resulted in increased insulin level and decreased insulin resistance.

**Effect of RSV on serum SOD activity**

**Fig 1:**
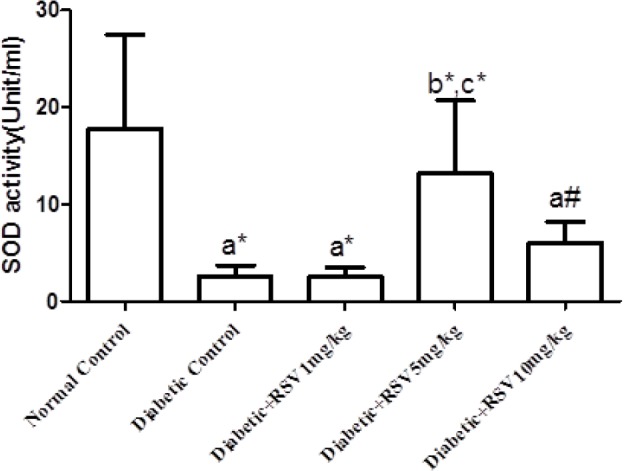
Effect of different doses of RSV on SOD activity in studied groups. Normal control: healthy control group; Diabetic Control: diabetic untreated group; Diabetic+RSV 1 mg/kg: diabetic group treated with 1 mg/kg.bw/day of RSV; Diabetic+RSV 5 mg/kg: diabetic group treated with 5 mg/kg.bw/day of RSV; Diabetic+RSV 10 mg/kg: diabetic group treated with 10 mg/kg.bw/day of RSV. a: compared with normal control group; b: compared with diabetic control group; c: compared with diabetic rats that received RSV (1 mg/kg.bw/day).*: P <0.05; #: P= 0.001

Figure 1 indicates the effect of treatment with RSV on serum SOD activity. The serum SOD activity in untreated diabetic group (diabetic control) and diabetic groups treated with 1 and 10 mg/kg.bw/day of RSV decreased statistically in comparison with the normal control group. Furthermore, treatment with 5 mg/kg.bw/day of RSV improved the SOD activity in comparison with the diabetic control group.

**Table 1:** The sequence of primers used in RT-PCR

**Gene**	**Primers 5’ → 3’**	**GC%**	**Tm (** ^o^ **C)**	**Amplicon size (bp)**
*FOXO1* NM_001191846	F: CGAGTGGATGGTGAAGAGTG R: CGAATAAACTTGCTGTGTAGGG	55.00 44.50	55.00 54.90	114
*FOXO3a* NM_001106359	F:CTCCCGTCAGCCAGTCTATG R: GCTTAGCACCAGTGAAGTTCC	60.00 52.40	56.60 55.50	270
*18S RNA* NM_046237.1	F: GTAACGCGTTGAACCCCATT R: CCATCCAATCGGTAGTAGCG	54.80 54.40	64.50 64.20	151

**Table 2:** Effect of different doses of resveratrol on glucose, insulin level, and insulin resistance (HOMA)

**Diabetic+RSV** **10 mg/kg**	**Diabetic+RSV** **5 mg/kg**	**Diabetic+RSV** **1 mg/kg**	**Diabetic Control**	**Normal Control**	
90.5±7.23	84.1±11	93.2±11.4	95.62±11.34	87.3±10.6	**Glucose D0 (mg/dl)**
190.33±68.63^b^*	192.5±84.8^b^*	273.37±77.57^a^*	303.3±92.1^a^*	92±10.8	**Glucose D37 (mg/dl)**
9.83±0.86^b^*, c*	9.52±1.05^b^*	8.13±0.97^a^*	7.23±1.15^a^*	11.17±1.06	**Insulin (U/ml)**
4.77±1.84^b^*,^c^*	3.73±2.01^b^*	5.52±1.92^a^*	5.46±2.09^a^*	2.51±0.37	**HOMA**

a : compared with normal control group;

b : compared with diabetic control group;

c : compared with diabetic rats that received RSV (1 mg/kg.bw/day); D0: before induction ofT2DM; D37: after completion of treatment period.

* : P <0.05.

**Fig 2:**
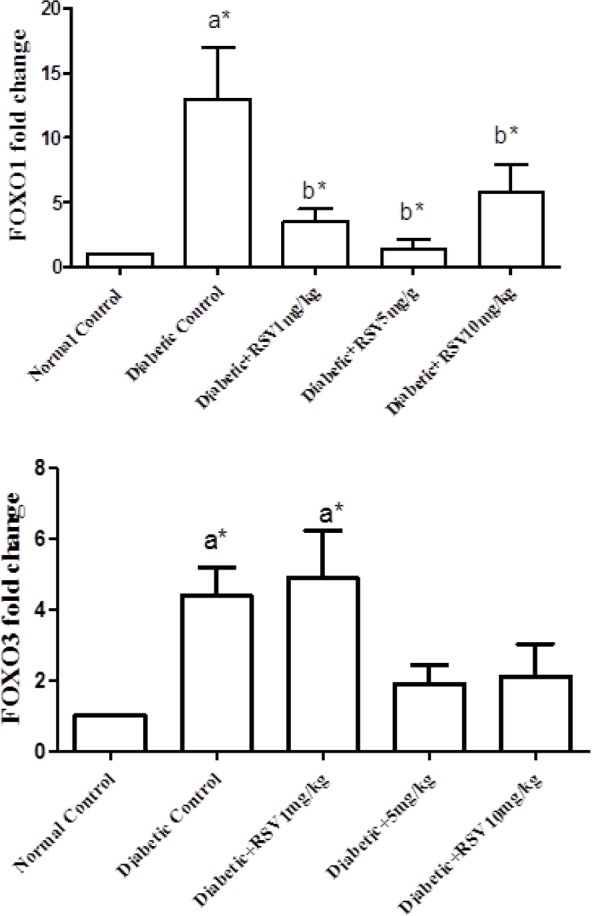
Effect of different doses of RSV on FOXO1 (A) and FOXO3a (B) genes expression in adipose tissue of the studied groups. Normal control: healthy control group; Diabetic Control: diabetic untreated group; Diabetic+RSV 1 mg/kg: diabetic group treated with 1 mg/kg.bw/day of RSV; Diabetic+RSV 5 mg/kg: diabetic group treated with 5 mg/kg.bw/day of RSV; Diabetic+RSV 10 mg/kg: diabetic group treated with 10 mg/kg.bw/day of RSV. a: compared with normal control group; b: compared with diabetic control group. *: P <0.05

**Table 3:** Correlation between the studied parameters

	**FOXO1**	**FOXO3a**	**HOMA (insulin resistance)**
**SOD activity**	R=- 0.396 P=0.062	R=- 0.478 P=0.21	R=- 0.459 P=0.028

SOD: Superoxide dismutase

**Effects of RSV on** ***FOXO1*** **and** ***FOXO3a*** **expression in adipose tissue**

As shown in Fig 2a, the mRNA level of *FOXO1* in untreated diabetic group significantly increased in comparison with the normal group, and treatment with different doses of RSV reduced the mRNA level of *FOXO1*. Fig 2b indicates that the mRNA level of *FOXO3a* increased significantly in untreated diabetic rats and diabetic group treated with 1 mg/kg.bw/day RSV in comparison with the normal group. However, treatment with 5 and 10 mg/kg.bw/day doses of RSV decreased the *FOXO3a* expression and brought it back mostly to the normal level.

**Correlation analysis**

As indicated in Table 3, a significant negative correlation was observed between *FOXO3a *expression in adipose tissue and serum SOD activity (R= -0.478; P= 0.021). In addition, a negative correlation was detected between *FOXO1* expression in adipose tissue and serum SOD activity (R= -0.396; P= 0.062). Moreover, a similar significant negative correlation was observed between serum SOD activity and insulin resistance (HOMA) (R= -0.459; P=0.028).

## Discussion

Due to the importance of oxidative stress in diabetes and insulin resistance, and the application of RSV in attenuating oxidative stress, we investigated the effects of different doses of RSV (1, 5 and 10 mg/kg.bw) on blood glucose, insulin level and insulin resistance, oxidative stress marker (SOD) in the sera of T2DM rats. In addition, we examined the RSV effect on *FOXO1* and *FOXO3a *gene expression in adipose tissue of these animals. Our findings indicated that RSV had significantly decreased blood glucose level, increased insulin level, and improved the insulin sensitivity. RSV resulted in an increased serum SOD activity and caused a decrease of *FOXO1* and *FOXO3a *expression in adipose tissue of rats with T2DM

Oxidative stress induced by hyperglycemia plays a key role in the development of DM complications such as cardiovascular disease (3, 4). Oxidative stress may lead to initiation and progression of insulin resistance through different mechanisms (5, 20, 21), which consequently result into diabetic complications such as coronary heart disease (4). Adipose tissue is an insulin-sensitive organ that plays an important role in oxidative stress and subsequently on insulin resistance. Therefore, in the present study adipose tissue was selected as the target tissue. Moreover, different chemical and herbal agents have been examined for their ameliorating effects on oxidative stress and subsequent attenuation of insulin resistance (22). RSV is a polyphenolic compound, mostly known as an antioxidant and hypoglycemic agent (23). Therefore, understanding the mechanism of RSV action provides beneficial information about its antioxidant and hypoglycemic effects.

In previous reports, we indicated that RSV has a powerful hypoglycemic effect and antioxidant properties (24, 25). Our results indicated that treatment with RSV caused blood glucose level decrease in diabetic rats in comparison with the untreated diabetic group. This result is in agreement with the findings of Rivera et al. and Palsamy et al. (26, 27). Additionally, our data indicated that RSV increased serum SOD activity, ameliorated oxidative stress, increased insulin concentration, and decreased insulin resistance. Oxidative stress can result in insulin resistance by phosphorylating serine residues in insulin receptor, which subsequently inhibits the phosphorylation of the tyrosine residues, and ultimately causes insulin receptors inactivation and insulin resistance (21). Also, the activation of *FOXO* transcription factors under hyperglycemic conditions may lead to insulin resistance. These transcription factors have a critical role in oxidative stress and insulin resistance (5, 28). The activities of these transcriptional factors are regulated by acetylation and phosphorylation. Deacetylation of these factors by SIRTs leads to their phosphorylation and inactivation (29). In DM, the activity of SIRTs decreases (13, 30, 31), and leads to insulin resistance (31). Finally, during diabetes SIRTs deacetylase activity decreases and results in FOXO deacetylation decrease, and consequently increasing *FOXO* expression that ultimately causes oxidative stress (8, 9). Our findings indicated that *FOXO3a *expression increased in adipose tissue of untreated diabetic rats, and treatment with RSV ameliorated* FOXO3a* expression in adipose tissue. Additionally, correlation analysis showed a significant negative correlation between *FOXO3a* expression and the activity of SOD, and a negative correlation between SOD activity and HOMA. Moreover, as the expression of *FOXO3a* increased, the activity of SOD decreased and caused increasing insulin resistance. It is possible that treatment with RSV attenuates the *FOXO3a* expression by affecting SIRTs, which may lead to the increase of the SOD activity, and ameliorate insulin resistance especially in rats that received 5 mg/kg of RSV. *FOXO1* is another member of *FOXO *gene family, which has a main function in glucose hemostasis and insulin resistance. During fasting state, *FOXO1* is dephosphorylated and stimulates gluconeogenesis (32, 33). Additionally, due to reduction of SIRT activity and its expression in DM, *FOXO1* is acetylated and activated which leads to hyperglycemia. Hyperglycemia can cause excess ROS production, and induction of oxidative stress may ultimately lead to insulin resistance (32, 34-36). Previous studies showed that during adipose tissue *FOXO1* gene silencing in transgenic mice, insulin resistance decreased and glucose tolerance improved (36, 37). Additionally, Kim et al. showed that betaine inhibited the activity of *FOXO1* and caused oxidative stress amelioration (28). Therefore, it can be concluded that insulin resistance can be improved by targeting the expression of *FOXO1* in the adipose tissue by specific agents. The results of the present study indicated that *FOXO1 *expression increased in untreated diabetic rats. Therefore, it could be hypothesized that RSV by exerting a beneficial effect on *FOXO1 *expression resulted in decreasing blood glucose level, improving subsequent oxidative stress and ameliorating insulin resistance. Furthermore, this hypothesis was strengthened by our correlation analysis that indicated a negative correlation between *FOXO1* expression and SOD activity. Our hypothesis needs to be confirmed by further studies.

The obtained results indicated the potential hypoglycemic and antioxidant properties of RSV that consequently ameliorated insulin resistance in type 2 diabetic rats. These RSV effects were implemented through attenuation of *FOXO* gene expression in adipose tissue of the animal model of type 2 diabetes.
